# Bromide of Lithium

**Published:** 1877-10

**Authors:** 


Bromide of Lithium.—Bromide of lithium, introduced into therapeutics some years since, is doubly valuable since it acts with equal energy as a sedative and a lithotriptic. In fact, its richness in bromine, 91.95 per cent., is very much superior to that of the other bromides, and its 8.05 per cent of lithium is sufficient to neutralize a considerable quantity of uric acid, as one part of lithium neutralizes four parts of uric acid.

Thanks to this double action, demonstrated both in the Paris hospitals and in civil practice, the bromide of lithium is vulgarly prescribed for the neuroses on the one hand, and on the other for the various manifestations of the uric diathesis. Experience, indeed, has demonstrated its powerful effects in epilepsy, chorea, insomnia, hypochondria, and, in the diverse forms of the uric diathesis, such as gravel, nephritic colic, gout and diabetes.

In these last affections which are accompanied by the symptom pain, as in nephritic colic and gout, bromide of lithium, besides its lithontriptic action, acts also as a sedative and calms in a few hours the suffering of the patients.

In this special relation the bromide of lithium is a most valuable remedy in therapeutics and cannot be too much recommended to physicians.—*Presse Med. Belge* (*La France Medicale.*)—*The Journal of Nervous and Mental Diseases.*

